# The Effects of Eccentric Training on Undulatory Underwater Swimming Performance and Kinematics in Competitive Swimmers

**DOI:** 10.5114/jhk/175824

**Published:** 2024-05-17

**Authors:** Jesús J. Ruiz-Navarro, Óscar López-Belmonte, Francisco Cuenca-Fernández, Ana Gay, Raúl Arellano

**Affiliations:** 1 Aquatics Lab, Department of Physical Education and Sports, Faculty of Sport Sciences, University of Granada, Granada, Spain.; 2 Department of Sports and Computer Sciences, Universidad Pablo de Olavide, Seville, Spain.

**Keywords:** assessment, biomechanics, speed, swimmers, dolphin kick

## Abstract

This study aimed to evaluate the effects of a five-week training program on undulatory underwater swimming (UUS) in swimmers and to compare the specific effects prompted by two different training protocols on UUS performance and kinematics. Swimmers (n = 14) were divided into in-water only (WO) (18.61 ± 2.62 years, FINA points: 507 ± 60) and water + dry-land training groups (with conical pulleys) (WD) (18.38 ± 2.67 years, FINA points: 508 ± 83). Three countermovement jumps (CMJ) and three maximal UUS trials were performed before and after a five-week training period. The training program comprised 14 × 30-min sessions. The WO group repeated the same 15-min block twice, while the WD group performed one block of 15 min in the water and the other block on land performing lower limb exercises with conical pulleys. Seven body landmarks were auto-digitalized during UUS by a pre-trained neural network and 21 kinematic variables were calculated. The level of statistical significance was set at p < 0.05. Significant time × group interaction in favour of the WD group was observed for mean vertical toe velocity (p = 0.035, $\eta_{p}^{2}$ = 0.32). The WD group experienced enhancements in mean and maximum underwater velocity, kick frequency, maximum shoulder angular velocity, as well as mean and maximum vertical toe velocity (p < 0.05). The WO group exhibited an enhancement in CMJ height (p < 0.05). In conclusion, UUS performance was improved in adolescent swimmers after five weeks of specific training, only when combining water and conical pulley exercises. Coaches should include dry-land specific lower limb exercises in addition to in-water training to improve UUS performance.

## Introduction

In swimming, having well-developed acyclic phases (i.e., start and turns) is considered an essential prerequisite to yield high performance in major international events (Arellano et al., 2022; Cuenca-Fernández et al., 2022). These acyclic phases are often divided into subsections for in-depth analysis, such as diving, wall push-off, underwater, and breakout (Gonjo and Olstad, 2021). Among these subsections, start and turn performances clearly rely on the optimization of the underwater phase (Mason and Cossor, 2001). The prominence of this phase is frequently observed in major events, where most of swimmers try to reach the limited 15 m after each wall as an important contribution to the overall performance (Veiga and Roig, 2016). Therefore, coaches should consider that any improvements within the underwater phase would lead to an enhancement of the start and turn performances, thus having a crucial impact on the overall race success.

Swimmers propel themselves forward throughout the underwater phase by performing undulatory underwater swimming (UUS), also known as a “dolphin kick”, even in breaststroke events, where swimmers are allowed to perform one kick with a high contribution to the underwater propulsion. UUS consists of performing body undulations while holding a streamline body position with arms outstretched and held together over the head (Ruiz-Navarro et al., 2022b). The propulsion is generated in a “whip-like” action and this “body wave” travels caudally throughout the body, resulting in a leg-dominated technique (Higgs et al., 2017). UUS velocity can be enhanced by increasing the magnitude of the propulsive impulse relative to the active drag experienced, hence, equal velocities can be reached in a number of different ways (Connaboy et al., 2016).

Among all the kinematic variables, kicking frequency, cycle length, joint amplitudes, range of motion (ROM), and maximum angular velocity seem to be related to UUS technique and performance (Connaboy et al., 2010; Lyakh et al., 2014, 2016; Ruiz-Navarro et al., 2022b; Veiga et al., 2024). Moreover, the fact that specific activation of lower limb muscles enhances UUS performance (Crespo et al., 2021) and that the use of lower body strength exercises enhances other swimming leg-dominated techniques, such as flutter kicking (Mookerjee et al., 1995) and the swimming start (García-Ramos et al., 2016), support the likely role of muscle strength in UUS performance (Willems et al., 2014). Several previous studies have focused on finding the strongest predictors of UUS performance (Atkinson et al., 2014; Bielec et al., 2010, 2013; Connaboy et al., 2016; Higgs et al., 2017; Ruiz-Navarro et al., 2022b; Tanaka et al., 2022). Nevertheless, the results were variable partly due to inconsistencies in the kinematic variables measured (Veiga et al., 2022) or because of swimmers using different techniques when performing maximal UUS (Connaboy et al., 2016).

The effects of UUS in-water training programs have been mostly studied in youth swimmers (Collard et al., 2013; Helmy, 2013; Ruiz-Navarro et al., 2021) as the optimum age for learning swimming techniques ranges between 7 and 12 years old (Navarro et al., 2003). However, as well as it happens in the rest of the strokes, swimmers experience the dramatic improvement in UUS velocity throughout the adolescence (Nikolaidis, 2012). Hence, it remains unclear how a period of UUS specific training might affect performance in this group of swimmers. Furthermore, as the propulsive forces yielded while swimming rely on aquatic-based strength, directly related to dry-land strength (Ruiz-Navarro et al., 2020; 2022c), the specific effects of lower limb strength training on UUS performance are unknown. Therefore, this study aimed 1) to evaluate the effects of a five-week UUS training program in adolescent swimmers, and 2) to compare the specific effects prompted by two different training protocols on UUS performance and kinematics. It was hypothesized that UUS performance would be enhanced in both training groups, especially when including dry-land training composed only of conical pulley exercises.

## Methods

### Participants

Nineteen (10 males and 9 females) trained swimmers (Mckay et al., 2022), competing mainly in 50- and 100-m events, volunteered to participate in the current study. Swimmers performed six in-water and four dry-land training sessions per week in the same squad following the same training regimen under the direction of the same coach, with more than two years of conical pulley exercise experience (i.e., concentric and eccentric training). From the initial 19 participants that were randomly assigned to each group, two females and two males did not meet the study criteria (i.e., took part in less than 85% of the training sessions). Moreover, one female swimmer dropped out due to an injury (not related with the study). Hence, a total of 14 swimmers, eight males and six females, completed the whole training program and were finally included in the analysis. The WO group was composed of four males and three females (18.6 ± 2.6 years, 65.2 ± 8.7 kg of body mass, 169.8 ± 5.6 cm of body height, and 50-m front crawl International Swimming Federation [FINA] points: 507 ± 60, performance level 4 (Ruiz-Navarro et al., 2022d)) and the WD comprised also four males and three females (18.4 ± 2.6 years, 63.7 ± 7.4 kg of body mass, 172.7 ± 7.3 cm of body height, and 50-m front crawl FINA points: 508 ± 83, performance level 4 (Ruiz-Navarro et al., 2022d)). The protocol was explained to swimmers and their parents (swimmers’ under 18 years), prior to signing an informed written consent form. The study was conducted according to the Code of Ethics of the World Medical Association (Declaration of Helsinki) and the protocol was approved by the University of Granada ethics committee (protocol code 852; approval date: 14 February 2014).

### Measures

The countermovement jumps (CMJs) were analyzed using MyJump 2 (Balsalobre-Fernández et al., 2015). From the five CMJ analyzed, the highest and the lowest CMJ heights were removed, and the mean CMJ height of the three remaining trials was calculated (Perez-Olea et al., 2018).

For UUS analysis bilateral symmetry was assumed (Connaboy et al., 2010) and only the right side was examined using a trained Neural Network in DeepLabCut^TM^. The training procedures were conducted following the methods employed by Papic et al. (2020) on a manually digitized subset of 400 frames taken from the UUS trials. The mean test error between manually digitized body landmarks and the neural network was 2.08 pixels or 5.5 mm. The “Cinalysis” software (Elipot et al., 2010) was used to compute the calibration coefficients by applying a 2D direct linear transformation with a calibration plane (2.05 x 1.60 m) containing 37 calibration points in Matlab 2016 (MathWorks Inc., Natick, Mass., USA). The calibration error was assessed as the reprojection error, where the root-mean-square error (RMSE) of the reconstructed calibration marker positions was for the x- and y-axis coordinates 3.1 mm and 2.9 mm, respectively. Per video recording, two full cycles were digitized. In addition, 15 frames before and after the start and the end of the two kick cycles were also digitized to prevent minimization of the data during smoothing and subsequent calculation of time derivatives (Vaughan, 1982). A fourth-order low pass Butterworth filter with a cut-off frequency of 6 Hz was employed to smooth the data (Ruiz-Navarro et al., 2021).

Using the methods employed by Connaboy et al. (2010), a total of 21 kinematic variables already identified as important in UUS were calculated for each kick cycle: mean, maximum, and minimum swimming velocity (denoted as: mean U, max U, and min U, respectively), cycle length, kick frequency, vertical joint center amplitudes of the wrist, the shoulder, the hip, the knee, and the ankle, 5^th^ metatarsal phalangeal joint, maximum angular velocities of the shoulder, the hip, the knee, and the ankle, joint ranges of movement of the shoulder, the hip, the knee, and the ankle, mean and maximum vertical toe velocity. The calculation of variables was performed for each cycle in Python 3.9.

### Design and Procedures

A pre/post testing design was conducted with an intervention carried out over five weeks. The length of the training period and sessions were determined on the basis of the needs of the swimmers’ coach in relation to the competition calendar and the general training regime. Swimmers were evaluated before (PRE) and after (POST) the five-week training period. The intervention period took place during the second macrocycle of the season, ending right before the beginning of the taper. During this period swimmers followed the training programme set by their coach. Standard methods were used to calculate and categorise swimming training loads (defined using training units) using the five-zone system proposed by Mujika et al. (1996). The mean weekly volume and training units were 32160 + 4570 m and 51.93 + 9.72, respectively. Swimmers were randomly allocated into two groups: an only water group (WO), which conducted all the exercises in the water and a water + dry-land group (WD), which performed half of the time in the water and half on land during each session.

To avoid a possible learning effect, swimmers were familiarized with the experimental procedures before the intervention. In both PRE and POST conditions, testing was performed on the same time of the day to avoid possible biases due to circadian variation (Atkinson and Reilly, 1996). Furthermore, swimmers were instructed to refrain from intense exercise and/or vigorous physical activity and to abstain from stimulant beverages consumption 24 h before each testing session. The intervention included a total of 14 sessions of 30 min each that were part of the regular swimming training session. Throughout the intervention, swimmers were requested to attend at least 85% of the sessions (i.e., 12 sessions) and to follow the whole training program set by their coach.

In accordance with the swimmers’ coach, the training protocol comprised firstly two weeks of four sessions per week (Monday, Wednesday, Friday, and Saturday) and three weeks of two sessions per week (Monday and Wednesday). The training protocol was designed following the procedures employed by Ruiz-Navarro et al. (2021) dividing the exercises in five groups (“body awareness”, “gliding”, “gliding + propulsion”, “propulsion”, and “speed”). Since all participants were skilled swimmers and the alignment and the position of the swimmers’ body were correctly performed (observed by a biomechanic researcher), the training protocol focused on “gliding + propulsion”, “propulsion”, and “speed” exercises. The contents of each session progressed in difficulty or intensity over the five training weeks. The whole protocol is available in the supplementary material.

**Figure 1:**
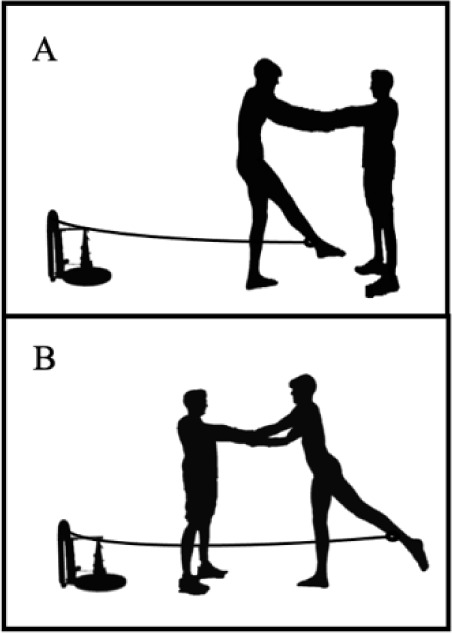
Lower limb exercises on land using conical pulleys. *A: downbeat action simulation (i.e., hip flexion + knee extension); B: upbeat action simulation (hip extension + knee flexion)*.

To make sure that both groups performed the same exercises and with similar intensity, each UUS training session was divided into two identical blocks of 15 min and a researcher attended all the training sessions to ensure that the training protocol was properly performed. Hence, the WO group performed the two 15-min blocks in the water, while the WD group performed one block of 15 min in the water and the other one on land using conical pulleys (RSP conic, Pontevedra, Spain). The dry-land exercises with conical pulleys were performed unilaterally (i.e., first one leg and then the other one). Standing on their feet and clinging to a partner, swimmers had to perform the downbeat action simulation (i.e., hip flexion + knee extension) or the upbeat action simulation (hip extension + knee flexion) (Figure 1). During the training period, the masses used in the conical pulleys varied, being the moment of inertia: 531.39 kg/cm^2^ in sessions 1–4 (0 masses added); 635.13 kg/cm^2^ in sessions 5–8 (2 masses added); and 738.86 kg/cm^2^ in sessions 9–14 (4 masses added). The conical pulley exercises were carried out alternately each day (e.g., Monday: downbeat action simulation, Wednesday: upbeat action simulation). The whole protocol is specified in the supplementary material.

**Figure 2:**
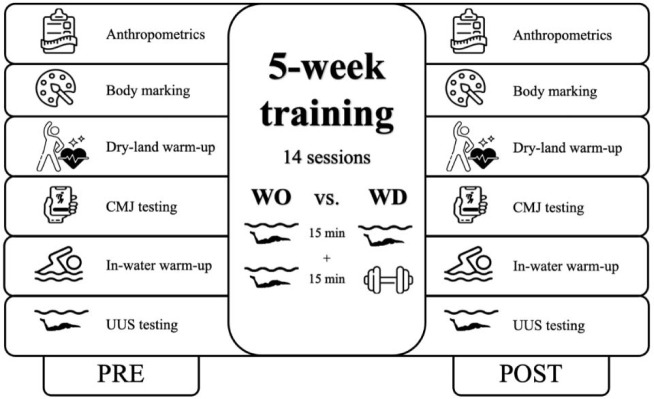
Study design and evaluations conducted. *CMJ: countermovement jump; UUS: undulatory underwater swimming. PRE: before the five-week training period; POST: after the five-week training period*.

During both testing sessions (i.e., PRE and POST) (Figure 2), anthropometric measurements were conducted by the same researcher using a stadiometer (Seca 799, Hamburg, Germany) at swimmers’ arrival to the facilities. Participants were then marked with a 3-cm-diameter circle of black oil-based hypoallergenic body paint at the styloid process of the ulna, the head of the humerus, the greater trochanter of the femur, the lateral epicondyle of the femur, the lateral malleolus of the fibula, and the 5^th^ metatarsal phalangeal joint of the foot (5^th^ MPJ) of the right side of the body. These specific points represented the joint centers of the wrist, the shoulder, the hip, the knee, and the ankle and the most distal point of the foot, respectively (Naemi and Sanders, 2008). Subsequently, a standardized warm-up of dry-land exercises meant to activate and mobilize the core and lower limbs before performing maximal effort tests was conducted (McLeod, 2009; Ruiz-Navarro et al., 2021): ankles, knees, hips, shoulder joint mobility, 2 × 10 repetitions of squats with 30-s rest intervals, 2 × 10 repetitions of the lunge with 30-s rest intervals, 2 × 30 s of planks with 15-s rest intervals, 2 × 30 s of the bird dog with 15-s rest intervals, and 3 submaximal CMJs (from an upright position with hands akimbo, swimmers bent the lower limbs to a self-selected depth and jumped without pausing). Following the dry-land warm-up, five maximal CMJs with no arm swing and 1 min of the rest interval in between were recorded with an iPhone X (apple inc., California, USA) high-velocity camera in an adjacent room to the pool by the same researcher during both testing days.

Swimmers then entered a 25-m swimming pool (25 × 16.5 m; 28 and 27.8ºC water temperature, 31.0 and 30.6ºC air temperature and 32 and 44% humidity in the PRE and POST condition, respectively) and performed an in-water warm-up comprising a 400-m swim, a 100-m pull, a 100-m kick, 4 × 50-m progressive speed, 4 submaximal underwater trials familiarizing with the procedures, and 200-m easy swim (Ruiz-Navarro et al., 2020). The UUS assessment was performed in the same pool and consisted of three maximum 15-m trials with 3 min of total recovery in between (Higgs et al., 2017). Each trial was performed at 1-m depth beginning with swimmers pushing prone from the wall at 1-m depth to remove wave drag effects (Vennell et al., 2006). Swimmers were asked to maintain the depth at 1 m throughout the 15 m otherwise they would be requested to perform an additional trial. Moreover, to avoid the velocity obtained during kicking being affected by the push-off from the wall, swimmers were asked to start kicking as soon as possible (Arellano et al., 2002). One stationary underwater camera (GoPro HERO 9, 60Hz, 2.7K, California, USA) was set up at 7.5 m from the starting wall and 1 m below the surface with the optical axes perpendicular to the direction of swimming, recording the area between 5 and 10 m. This area ensured that two complete kick cycles per trial were recorded (Connaboy et al., 2010). Hence, a total of six cycles (two cycles per trial) were captured for analysis guarantying a representative and reliable account of the UUS kinematics (Connaboy et al., 2010).

### Statistical Analysis

The data are expressed as mean ± standard deviation (SD). Normality and homogeneity of variance across groups (WO vs. WD) of the data sets were verified using the Shapiro-Wilk and Levene’s tests, respectively. An independent *t*-test was used to compare swimmers’ characteristics between groups. A 2 × 2 (group: WO, WD, and time: PRE, POST) repeated measures analysis of variances (ANOVA) was performed for each variable and the Bonferroni post-hoc test was used. The effect size for main effects was expressed as partial eta squared ($\eta_{p}^{2}$). Likewise, the effect size was calculated using Cohen’s *d* to estimate the magnitude of the training effect on the analysed variables within each group. In this case, the effect size was categorized as follows: small if 0 ≤ |*d*| ≤ 0.5, medium if 0.5 < |*d*| ≤ 0.8, and large if |*d*| > 0.8 (Cohen, 1988). All the statistical procedures were performed using SPSS (version 24.0, IBM) with the level of statistical significance set at *p* < 0.05.

## Results

At baseline, there were no significant differences between groups considering age, body height, body mass, and 50 m front crawl FINA points (*p* > 0.05).

Significant time × group interaction in favour of the WD group was observed for mean vertical toe velocity (*p* = 0.035, $\eta_{p}^{2}$ = 0.32), whereas there was no time × group interaction for the rest of the variables (*p* > 0.05). Training resulted in a main effect of time in Mean U (*p* = 0.003, $\eta_{p}^{2}$ = 0.53), Max U (*p* = 0.005, $\eta_{p}^{2}$ = 0.49), kick frequency (*p* = 0.033, $\eta_{p}^{2}$ = 0.32), maximum shoulder angular velocity (*p* = 0.013, $\eta_{p}^{2}$ = 0.41), maximum knee angular velocity (*p* = 0.028, $\eta_{p}^{2}$ = 0.34), mean vertical toe velocity (*p* = 0.035, $\eta_{p}^{2}$ = 0.32) and maximum vertical toe velocity (*p* = 0.035, $\eta_{p}^{2}$ = 0.32). This main effect of time was only significant in the WD group (Table 1). Mean value differences between PRE and POST, relative change, and effect size for all variables are reported in Table 1.

**Table 1:** Mean ± standard deviation, changes in undulatory underwater swimming performance and kinematics and countermovement jumps from PRE to POST training for each group.

Variable	Group	PRE-test	POST-test	Difference [95% CI]; Δ%	*p*-value	Effect size
Mean U (m/s)	WO	1.53 ± 0.14	1.55 ± 0.16	0.02 [−0.02, 0.06]; 1.3%	0.295	0.13, small
	WD	1.50 ± 0.17	1.58 ± 0.17	0.08 [0.04, 0.12]; 5.0%	0.001^*^	0.47, small
Max U (m/s)	WO	1.85 ± 0.18	1.89 ± 0.19	0.04 [−0.03, 0.12]; 2.4%	0.212	0.22, small
	WD	1.80 ± 0.15	1.92 ± 0.18	0.12 [0.05, 0.19]; 6.7%	0.004^*^	0.72, medium
Min U (m/s)	WO	1.17 ± 0.13	1.16 ± 0.16	−0.10 [−0.50, 0.70]; −0.8%	0.723	0.07, small
	WD	1.11 ± 0.23	1.14 ± 0.21	0.03 [−0.02, 0.09]; 3.3%	0.203	0.14, small
Cycle length (m)	WO	0.76 ± 0.08	0.77 ± 0.10	0.00 [−0.03, 0.04]; 0.4%	0.873	0.11, small
	WD	0.72 ± 0.01	0.70 ± 0.07	−0.02 [−0.05, 0.02]; −2.7%	0.273	0.23, small
Kick frequency (Hz)	WO	2.01 ± 0.26	2.04 ± 0.33	0.03 [−0.09, 0.15]; 1.4%	0.613	0.10, small
	WD	2.09 ± 0.10	2.25 ± 0.16	0.16 [0.04, 0.27]; 7.5%	0.014^*^	1.20, large
Wrist amplitude (m)	WO	0.06 ± 0.01	0.07 ± 0.02	0.01 [−0.01, 0.02]; 9.4%	0.402	0.39, small
	WD	0.08 ± 0.03	0.09 ± 0.04	0.01 [−0.01, 0.02]; 13.1%	0.147	0.28, small
Shoulder amplitude (m)	WO	0.06 ± 0.01	0.06 ± 0.01	0.00 [−0.01, 0.01]; 9.7%	0.142	0.36, small
	WD	0.06 ± 0.01	0.06 ± 0.01	0.00 [−0.01, 0.01]; 10.3%	0.114	0.44, small
Hip amplitude (m)	WO	0.13 ± 0.01	0.14 ± 0.02	0.01 [−0.01, 0.02]; 7.4%	0.107	0.54, medium
	WD	0.13 ± 0.03	0.13 ± 0.03	0.00 [−0.01, 0.01]; 2.0%	0.638	0.08, small
Knee amplitude (m)	WO	0.26 ± 0.03	0.26 ± 0.03	0.00 [−0.01, 0.02]; 1.7%	0.642	0.13, small
	WD	0.27 ± 0.04	0.27 ± 0.04	−0.00 [−0.02, 0.01]; −2.0%	0.529	0.14, small
Ankle amplitude (m)	WO	0.43 ± 0.04	0.42 ± 0.06	−0.01 [−0.04, 0.01]; −3.1%	0.371	0.25, small
	WD	0.41 ± 0.05	0.42 ± 0.04	−0.01 [ −0.03, 0.02]; −0.7%	0.830	0.07, small
5^th^ MPJ amplitude (m)	WO	0.56 ± 0.05	0.55 ± 0.06	−0.01 [−0.03, 0.02]; −1.0%	0.699	0.09, small
	WD	0.57 ± 0.05	0.56 ± 0.05	−0.01 [−0.03, 0.02]; −1.4%	0.511	0.14, small
Max shoulder angular velocity (º/s)	WO	177.58 ± 46.42	185.88 ± 37.76	8.29 [−0.833, 24.91]; 4.7%	0.298	0.20, small
	WD	179.76 ± 65.91	202.69 ± 69.63	22.92 [6.30, 39.55]; 12.8%	0.011^*^	0.34, small
Max hip angular velocity (º/s)	WO	463.02 ± 49.43	467.57 ± 59.56	4.54 [−55.47, 64.56]; 0.9%	0.872	0.08, small
	WD	481.70 ± 75.67	505.78 ± 79.83	24.08 [−35.93, 84.10]; 5.0%	0.399	0.31, small
Max knee angular velocity (º/s)	WO	707.47 ± 130.60	766.82 ± 126.42	59.35 [−2.70, 178.95]; 8.4%	0.180	0.46, small
	WD	720.43 ± 36.60	808.55 ± 116.70	88.12[−2.70, 178.95]; 12.2%	0.056	1.02, large
Max ankle angular velocity (º/s)	WO	677.81 ± 146.05	703.19 ± 177.08	25.38 [−60.95, 111.72]; 3.7%	0.533	0.16, small
	WD	531.87 ± 108.19	603.67 ± 129.73	71.80 [−14.53, 158.14]; 13.5%	0.095	0.60, medium
Shoulder ROM (º)	WO	23.82 ± 8.86	26.31 ± 3.95	2.03 [−0.09, 4.16]; −2.2%	0.060	0.60, medium
	WD	23.82 ± 8.86	24.96 ± 8.35	1.13 [−0.99, 3.26]; 4.7%	0.268	0.13, small
Hip ROM (º)	WO	44.57 ± 1.55	47.39 ± 5.93	2.83 [−1.16, 7.30]; 6.3%	0.192	0.66, medium
	WD	48.58 ± 6.46	46.89 ± 5.57	−1.69 [−6.16, 2.77]; −3.5%	0.425	0.28, small
Knee ROM (º)	WO	82.22 ± 5.72	81.58 ± 5.94	−1.23 [−4.59, 2.11]; −1.5%	0.437	0.04, small
	WD	81.55 ± 4.69	83.21 ± 4.53	−1.63 [−5.01, 1.69]; −1.9%	0.301	0.36, small
Ankle ROM (º)	WO	45.82 ± 5.37	45.82 ± 7.80	0.00 [−3.49, 3.49]; 0.0%	0.999	0.00, small
	WD	40.58 ± 5.87	42.99 ± 6.26	2.41 [−1.07, 5.90]; 5.9%	0.158	0.40, small
Mean toe vertical velocity (m/s)	WO	113.26 ± 14.89	112.86 ± 14.12	−0.393 [−4.78, 3.99]; −0.3%	0.849	0.03, small
	WD	119.70 ± 11.04	126.88 ± 12.69	7.17 [2.79, 11.56]; 6.0%	0.004^*^	0.60, medium
Max toe vertical velocity (m/s)	WO	403.62 ± 26.79	408.47 ± 30.01	4.85 [−15.24, 24.94]; 1.2%	0.608	0.17, small
	WD	414.65 ± 24.93	440.76 ± 24.89	26.11 [6.02, 46.19]; 6.3%	0.015^*^	1.05, large
CMJ_JH_ (m)	WO	0.31 ± 0.10	0.33 ± 0.11	0.02 [0.00, 0.04]; 7.5%	0.023^*^	0.22, small
	WD	0.36 ± 0.07	0.37 ± 0.06	0.01 [−0.01, 0.03]; 2.6%	0.360	0.12, small

WO: in-water only, WD: water + dry-land, Mean U: mean undulatory, underwater velocity Max U: maximum undulatory underwater velocity, Min U: minimum undulatory underwater velocity, MPJ: metatarsal phalangeal joint, ROM: range of motion, and CMJ_JH_: countermovement jump height. ^*^ significant differences

## Discussion

This study aimed to assess the effects of a five-week training protocol on UUS performance in adolescent swimmers and to compare the effects of two different training protocols on UUS performance and kinematics. Our hypothesis was partially confirmed as the current study indicated that adolescent swimmers’ UUS performance was enhanced after five weeks of specific training only when combining in-water and conical pulleys exercises. Therefore, these results provide relevant evidence to support the need of adding specific UUS strength exercises for the lower limbs within the swimming training program to obtain further development of adolescent swimmers’ performance.

The enhancement in UUS velocity could be achieved by either increasing propulsive force or decreasing the active drag experienced. Since the alignment and the position of the body were correct, as observed by a biomechanic researcher, the training protocol focused on improving the propulsive impulse. The enhancement in the propulsive forces could be achieved by either increasing muscle force or the ability to apply that force (Ruiz-Navarro et al., 2020, 2022a). In terms of muscle force, only the WO group reached greater CMJ_H_ after the training period, which might indicate that lower limb strength was not significantly developed by conical pulley training in the WD group (Table 1). Nevertheless, it is important to mention that the study of propulsion in UUS relies mainly on the analysis of the vortex, which has been positively related to vertical toe velocity (i.e., the higher vertical toe velocity the greater the propulsion) (Ungerechts et al., 2000). Indeed, our results showed a significant interaction for mean vertical toe velocity. The WD group increased the mean vertical toe velocity (6.0%), while the WO group showed almost identical results (−0.3%) after the training period, which might explain why UUS performance only improved in the WD group (Table 1). Hence, conical pulley exercises combined with in-water training enhanced mean vertical toe velocity and likely led to the Mean U and Max U improvement in the WD group (5.0% and 6.7%, respectively).

Often swimmers increase UUS velocity by increasing the kick frequency and reducing cycle length in a relatively lower proportion (Yamakawa et al., 2022). This process seems to require a period of adaptation since acute kick frequency changes are matched by cycle length reduction eliciting similar UUS velocity (Shimojo et al., 2014). However, even after five weeks of training, cycle length and kick frequency were similar in the WO group (Table 1). As an increase in kick frequency requires more internal work of locomotion (Zamparo et al., 2002), it is possible that swimmers in the WO group were not able to produce larger torque power that would enable them to reach higher kick frequency without compromising cycle length (Shimojo et al., 2014). Nevertheless, the WD group increased kick frequency after the five-week period, which together with the maintenance of cycle length (Table 1) explains the UUS performance improvement elicited by training. It is possible then, that WD swimmers were able to produce higher torque in the POST compared to the PRE condition. This fact is indeed in line with the greater vertical toe velocity observed in the WD group in the POST than in the PRE condition.

Cycle length and kick frequency are modulated by joint amplitude, joint angular velocity, joint ROM, and vertical toe velocity (Connaboy et al., 2016; Yamakawa et al., 2022). Altogether, these kinematic variables represent swimmers’ UUS technique, being therefore possible to attain the same UUS velocity in several different ways (Connaboy et al., 2016). For instance, some swimmers may seek to perform large undulatory movements maximizing propulsive impulse production, which would lead to higher joint amplitude, whereas other swimmers may perform smaller movements (i.e., lower joint amplitude and ROM) to produce a reduced amount of propulsive impulse, but instead an active drag reduction (Connaboy et al., 2016). Our results did not show significant changes in joint amplitude or ROM in any of the training groups (Table 1). Nevertheless, swimmers in the WD group experienced a significant improvement in the maximum shoulder angular velocity and a positive trend in the rest of the maximum joint angular velocities after the training period (Table 1). Thus, the resulting amount of positive trends obtained in all the maximum joint angular velocities (Connaboy et al., 2016) together with the increase in mean vertical toe velocity (Yamakawa et al., 2022), after the training period, likely induced the development of higher kick frequency in the WD group. Conversely, the WO group did not exhibit any change in joint angular velocities, which may explain the similar kick frequency observed. Hence, five weeks of only in-water training might not be sufficient to induce technical changes in adolescent swimmers or perhaps, the exercises included in our program should have been different to induce significant changes.

Certain limitations should be acknowledged. First, the small final sample analyzed, which could have reduced the statistical power. In fact, some of the variables showed a positive trend with a borderline *p*-value. Nevertheless, the inclusion of swimmers with a high percentage of missing sessions or swimmers from different squads could have introduced a risk of bias and negatively affected the results. Second, the lack of a specific control group or another resistance training program group would allow us to further analyze the effects of strength training. However, considering the sample size (as mentioned before, this was the largest sample possible), splitting it into three groups would have resulted in lower statistical power precluding from obtaining significant results. In addition, it was not ethical to restrain competitive swimmers from performing UUS training, especially during that period of the season.

## Conclusions

Five weeks of skill-specific training, including specific conical exercises, can induce performance enhancement in UUS, likely as a result of greater vertical toe velocity and kick frequency. However, only five weeks of skill-specific in-water training do not enhance UUS performance. Five weeks of in-water training could not be long enough or the exercises conducted in our research might not be adequate to induce changes in adolescent skilled swimmers. These results highlight that coaches should provide stimuli under dry-land conditions to improve UUS performance. Therefore, this aspect moves away from the more traditional trends that ensure that the development of the swimmer has to be exclusively in the water, and contributes to support the most current trends that comprehensively prioritize the development of swimmers, including a wide range of stimuli, both in the water and in the gym, to develop their physical and motor skills to the maximum.

## Figures and Tables

**Table:** 

SESSION 1, Monday, Week 1					
**Only Water (WO)**			**Water + Dry-land (WD)**		
Set	Rest	Exercise	Set	Rest	Exercise
4×50 m	@1’15”	Perform 2 powerful kicks and then glide for 2 s on the water surface using a snorkel in a prone streamline position with the arms outstretched and held together over the head. Repeat the action all the distance	4×50 m	@1’15”	Perform 2 powerful kicks and then glide for 2 s on the water surface using a snorkel in a prone streamline position with the arms outstretched and held together over the head. Repeat the action all the distance
8×15 m	@1’	Push off from the wall in a streamline position and glide. Right before stopping perform 5 powerful dolphin kicks and glide again as far as possible	8×15 m	@1’	Push off from the wall in a streamline position and glide. Right before stopping perform 5 powerful dolphin kicks and glide again as far as possible
4×50 m	@1’15”	Perform 2 powerful kicks and then glide for 2 s on the water surface using a snorkel in a prone streamline position with the arms outstretched and held together over the head. Repeat the action all the distance	4×10 rep each leg	@2’30”	Downbeat action simulation (i.e., hip flexion + knee extension) – 0 weights (moment of inertia: 531.39 kg/cm^2^)
8×15 m	@1’	Push off from the wall in a streamline position and glide. Right before stopping perform 5 powerful dolphin kicks and glide again as far as possible			

@: start every “X” time

**Table:** 

SESSION 2, Wednesday, Week 1					
**Only Water (WO)**			**Water + Dry-land (WD)**		
Set	Rest	Exercise	Set	Rest	Exercise
4×6 rep	@1’	Vertical kicks with fins and arms alongside the body	4×6 rep	@1’	Vertical kicks with fins and arms alongside the body
4×6 rep	@1’	Push from the bottom of the pool and perform vertical kicks with fins	4×6 rep	@1’	Push from the bottom of the pool and perform vertical kicks with fins
8×15 m	@1’	Reach 15 m in the shortest possible time using undulatory underwater swimming with fins	8×15 m	@1’	Reach 15 m in the shortest possible time using undulatory underwater swimming with fins
4×6 rep	@1’	Vertical kicks with fins and arms alongside the body	4×10 rep each leg	@2’30”	Upbeat action simulation (i.e., hip extension + knee flexion) – 0 weights (moment of inertia: 531.39 kg/cm^2^)
4×6 rep	@1’	Push from the bottom of the pool and perform vertical kicks with fins			
8×15 m	@1’	Reach 15 m in the shortest possible time using undulatory underwater swimming with fins			

@: start every “X” time

**Table:** 

SESSION 3, Friday, Week 1					
**Only Water (WO)**			**Water + Dry-land (WD)**		
Set	Rest	Exercise	Set	Rest	Exercise
4×50 m	@1’15”	Perform 2 powerful kicks and then glide for 2 s on the water surface using a snorkel in a prone streamline position with the arms outstretched and held together over the head. Repeat the action all the distance	4×50 m	@1’15”	Perform 2 powerful kicks and then glide for 2 s on the water surface using a snorkel in a prone streamline position with the arms outstretched and held together over the head. Repeat the action all the distance
8×15 m	@1’	Push off from the wall in a streamline position and glide. Right before stopping perform 7 powerful dolphin kicks and glide again as far as possible	8×15 m	@1’	Push off from the wall in a streamline position and glide. Right before stopping perform 7 powerful dolphin kicks and glide again as far as possible
4×50 m	@1’15”	Perform 2 powerful kicks and then glide for 2 s on the water surface using a snorkel in a prone streamline position with the arms outstretched and held together over the head. Repeat the action all the distance	4×10 rep each leg	@2’30”	Downbeat action simulation (i.e., hip flexion + knee extension) – 0 weights (moment of inertia: 531.39 kg/cm^2^)
8×15 m	@1’	Push off from the wall in a streamline position and glide. Right before stopping perform 7 powerful dolphin kicks and glide again as far as possible			

@: start every “X” time

**Table:** 

SESSION 4, Saturday, Week 1					
**Only Water (WO)**			**Water + Dry-land (WD)**		
Set	Rest	Exercise	Set	Rest	Exercise
8×25 m	@1’	Odd repetitions: swimming start with 15 m of the underwater phase Even repetitions: perform 5 kicks against the wall in a streamline body position and right after without breathing reach 10 m in the shortest possible time using undulatory underwater swimming	8×25 m	@1’	Odd repetitions: swimming start with 15 m of the underwater phase Even repetitions: perform 5 kicks against the wall in a streamline body position and right after without breathing reach 10 m in the shortest possible time using undulatory underwater swimming
2×100 m	@2’	Freestyle, increasing the number of underwater kicks every turn (4, 5, 6, 7 kicks per turn, respectively)	2×100 m	@2’	Freestyle, increasing the number of underwater kicks every turn (4, 5, 6, 7 kicks per turn, respectively)
8×25 m	@1’	Odd repetitions: swimming start with 15 m of the underwater phase Even repetitions: perform 5 kicks against the wall in a streamline body position and right after without breathing reach 10 m in the shortest possible time using undulatory underwater swimming	4×10 rep each leg	@2’30”	Upbeat action simulation (i.e., hip extension + knee flexion) – 0 weights (moment of inertia: 531.39 kg/cm^2^)
2×100 m	@2’	Freestyle, increasing the number of underwater kicks every turn (4, 5, 6, 7 kicks per turn, respectively)			

@: start every “X” time

**Table:** 

SESSION 5, Monday, Week 2					
**Only Water (WO)**			**Water + Dry-land (WD)**		
Set	Rest	Exercise	Set	Rest	Exercise
6×25 m	@1’10”	Push off from the wall in a streamline position at 1-m depth. Perform 2 powerful kicks and then glide for 2 s. Repeat the action all the lane along	6×25 m	@1’10”	Push off from the wall in a streamline position at 1-m depth. Perform 2 powerful kicks and then glide for 2 s. Repeat the action all the lane along
4×25 m	@1’15”	Push off from the wall in a streamline position at 1 m depth gliding until beginning to ascend to the surface, then kick all the lane along maintaining 1-m depth	4×25 m	@1’15”	Push off from the wall in a streamline position at 1-m depth gliding until beginning to ascend to the surface, then kick all the lane along maintaining 1-m depth
6×25 m	@1’10”	Push off from the wall in a streamline position at 1-m depth. Perform 2 powerful kicks and then glide for 2 s. Repeat the action all the lane along	4×8 rep each leg	@2’30”	Downbeat action simulation (i.e., hip flexion + knee extension) – 2 weights (moment of inertia: 635.13 kg/cm^2^)
4×25 m	@1’15”	Push off from the wall in a streamline position at 1-m depth gliding until beginning to ascend to the surface, then kick all the lane along maintaining 1-m depth			

@: start every “X” time

**Table:** 

SESSION 6, Wednesday, Week 2					
**Only Water (WO)**			**Water + Dry-land (WD)**		
Set	Rest	Exercise	Set	Rest	Exercise
4×6 rep	@1’	Vertical kicks with arms alongside the body	4×6 rep	@1’	Vertical kicks with arms alongside the body
4×8 rep	@1’	Push from the bottom of the pool and perform vertical kicks	4×8 rep	@1’	Push from the bottom of the pool and perform vertical kicks
8×15 m	@1’	Reach 15 m in the shortest possible time using undulatory underwater swimming	8×15 m	@1’	Reach 15 m in the shortest possible time using undulatory underwater swimming
4×6 rep	@1’	Vertical kicks with arms alongside the body	4×8 rep each leg	@2’30”	Upbeat action simulation (i.e., hip extension + knee flexion) – 2 weights (moment of inertia: 635.13 kg/cm^2^)
4×8 rep	@1’	Push from the bottom of the pool and perform vertical kicks			
8×15 m	@1’	Reach 15 m in the shortest possible time using undulatory underwater swimming			

@: start every “X” time

**Table:** 

SESSION 7, Friday, Week 2					
**Only Water (WO)**			**Water + Dry-land (WD)**		
Set	Rest	Exercise	Set	Rest	Exercise
4×50 m	@1’30”	Perform 2 powerful kicks with fins and then glide for 2 s on the water surface using a snorkel in a prone streamline position with the arms outstretched and holding a paddle over the head. Odd cycles with the paddle in a vertical position and even repetitions with the paddle in a horizontal position. Repeat the action all the distance	4×50 m	@1’30”	Perform 2 powerful kicks with fins and then glide for 2 s on the water surface using a snorkel in a prone streamline position with the arms outstretched and holding a paddle over the head. Odd cycles with the paddle in a vertical position and even repetitions with the paddle in a horizontal position. Repeat the action all the distance
2×100 m	@1’45”	Freestyle, perform undulatory underwater swimming during the 5 m in and 10 m out of every turn	2×100 m	@1’45”	Freestyle, perform undulatory underwater swimming during the 5 m in and 10 m out of every turn
4×50 m	@1’30”	Perform 2 powerful kicks with fins and then glide for 2 s on the water surface using a snorkel in a prone streamline position with the arms outstretched and holding a paddle over the head. Odd cycles with the paddle in a vertical position and even repetitions with the paddle in a horizontal position. Repeat the action all the distance	4×10 rep each leg	@2’30”	Downbeat action simulation (i.e., hip flexion + knee extension) – 2 weights (moment of inertia: 635.13 kg/cm^2^)
2×100 m	@1’45”	Freestyle, perform undulatory underwater swimming during the 5 m in and 10 m out of every turn			

@: start every “X” time

**Table:** 

SESSION 8, Saturday, Week 2					
**Only Water (WO)**			**Water + Dry-land (WD)**		
Set	Rest	Exercise	Set	Rest	Exercise
6×15 m	@1’15”	From the 5-m mark to the 20-m mark (without push-off) perform undulatory underwater swimming	6×15 m	@1’15”	From the 5-m mark to the 20-m mark (without push-off) perform undulatory underwater swimming
3×100 m	@2’15”	On the back perform kicks with the arms down at the side. Perform undulatory underwater swimming during 5 m in and 10 m out of every turn	3×100 m	@2’15”	On the back perform kicks with the arms down at the side. Perform undulatory underwater swimming during 5 m in and 10 m out of every turn
6×15 m	@1’15”	From the 5-m mark to the 20-m mark (without push-off) perform undulatory underwater swimming	4×10 rep each leg	@2’30”	Upbeat action simulation (i.e., hip extension + knee flexion) – 2 weights (moment of inertia: 635.13 kg/cm^2^)
3×100 m	@2’15”	On the back perform kicks with the arms down at the side. Perform undulatory underwater swimming during 5 m in and 10 m out of every turn			

@: start every “X” time

**Table:** 

SESSION 9, Monday, Week 3					
**Only Water (WO)**			**Water + Dry-land (WD)**		
Set	Rest	Exercise	Set	Rest	Exercise
4×25 m	@1’	On the water surface, move a teammate in a streamline position performing kicks with fins and snorkel in a prone streamline position with the arms outstretched and holding the colleagues' feet over the head	4×25 m	@1’	On the water surface, move a teammate in a streamline position performing kicks with fins and snorkel in a prone streamline position with the arms outstretched and holding the colleagues' feet over the head
4×25 m	@1’	Perform undulatory underwater swimming with fins in a prone streamline position with the arms outstretched and holding a paddle vertically over the head	4×25 m	@1’	Perform undulatory underwater swimming with fins in a prone streamline position with the arms outstretched and holding a paddle vertically over the head
4×15 m	@1’	Perform undulatory underwater swimming in a prone streamline position with the arms alongside the body	4×15 m	@1’	Perform undulatory underwater swimming in a prone streamline position with the arms alongside the body
4×25 m	@1’	On the water surface, move a teammate in a streamline position performing kicks with fins and snorkel in a prone streamline position with the arms outstretched and holding the colleagues' feet over the head	4×6 rep each leg	@2’30”	Downbeat action simulation (i.e., hip flexion + knee extension) – 4 weights (moment of inertia: 738.86 kg/cm^2^)
4×25 m	@1’	Perform undulatory underwater swimming with fins in a prone streamline position with the arms outstretched and holding a paddle vertically over the head			
4×15 m	@1’	Perform undulatory underwater swimming in a prone streamline position with the arms alongside the body			

@: start every “X” time

**Table:** 

SESSION 10, Wednesday, Week 3					
**Only Water (WO)**			**Water + Dry-land (WD)**		
Set	Rest	Exercise	Set	Rest	Exercise
8×15 m	@1’15”	Odd repetitions: swimming start with 15 m of the underwater phase Even repetitions: perform 5 kicks against the wall in a streamline body position and right after without breathing reach 15 m in the shortest possible time using undulatory underwater swimming	8×15 m	@1’	Odd repetitions: swimming start with 15 m of the underwater phase Even repetitions: perform 5 kicks against the wall in a streamline body position and right after without breathing reach 15 m in the shortest possible time using undulatory underwater swimming
2×100 m	@1’45”’	Freestyle, increase the number of underwater kicks every turn (6, 7, 8, 9 kicks per turn, respectively)	2×100 m	@1’45”’	Freestyle, increase the number of underwater kicks every turn (6, 7, 8, 9 kicks per turn, respectively)
8×15 m	@1’15”	Odd repetitions: swimming start with 15 m of the underwater phase Even repetitions: perform 5 kicks against the wall in a streamline body position and right after without breathing reach 15 m in the shortest possible time using undulatory underwater swimming	4×6 rep each leg	@2’30”	Upbeat action simulation (i.e., hip extension + knee flexion) – 4 weights (moment of inertia: 738.86 kg/cm^2^)
2×100 m	@1’45”’	Freestyle, increase the number of underwater kicks every turn (6, 7, 8, 9 kicks per turn, respectively)			

@: start every “X” time

**Table:** 

SESSION 11, Monday, Week 4					
**Only Water (WO)**			**Water + Dry-land (WD)**		
Set	Rest	Exercise	Set	Rest	Exercise
4×15”	@1’	Vertical kicks with fins with arms outstretched and held together above the head	4×15”	@1’	Vertical kicks with fins with arms outstretched and held together above the head
4×6 rep	@1’	Push from the bottom of the pool and perform vertical kicks	4×6 rep	@1’	Push from the bottom of the pool and perform vertical kicks
4×10”	@1’	Kick in a streamline body position against a teammate trying to displace him/her	4×10”	@1’	Kick in a streamline body position against a teammate trying to displace him/her
4×15”	@1’	Vertical kicks with fins with arms outstretched and held together above the head	4×8 rep each leg	@2’30”	Downbeat action simulation (i.e., hip flexion + knee extension) – 4 weights (moment of inertia: 738.86 kg/cm^2^)
4×6 rep	@1’	Push from the bottom of the pool and perform vertical kicks			
4×10”	@1’	Kick in a streamline body position against a teammate trying to displace him/her			

@: start every “X” time

**Table:** 

SESSION 12, Wednesday, Week 4					
**Only Water (WO)**			**Water + Dry-land (WD)**		
Set	Rest	Exercise	Set	Rest	Exercise
4× (3 rep + 10 m)	@1’30”	Push from the bottom of the pool, perform vertical kicks and then reach 10 m in the shortest possible time using undulatory underwater swimming with fins	4× (3 rep + 10 m)	@1’30”	Push from the bottom of the pool, perform vertical kicks and then reach 10 m in the shortest possible time using undulatory underwater swimming with fins
4×15 m	@2’	Competition between 2 swimmers per lane. Reach 10 m in the shortest possible time using undulatory underwater swimming with fins	4×15 m	@2’	Competition between 2 swimmers per lane. Reach 10 m in the shortest possible time using undulatory underwater swimming with fins
4× (3 rep + 10 m)	@1’30”	Push from the bottom of the pool, perform vertical kicks and then reach 10 m in the shortest possible time using undulatory underwater swimming with fins	4×8 rep each leg	@2’30”	Upbeat action simulation (i.e., hip extension + knee flexion) – 4 weights (moment of inertia: 738.86 kg/cm^2^)
4×15 m	@2’	Competition between 2 swimmers per lane. Reach 10 m in the shortest possible time using undulatory underwater swimming with fins			

@: start every “X” time

**Table:** 

SESSION 13, Monday, Week 5					
**Only Water (WO)**			**Water + Dry-land (WD)**		
Set	Rest	Exercise	Set	Rest	Exercise
4×6 rep	@1’	Vertical kicks with arms outstretched and held together above the head	4×6 rep	@1’	Vertical kicks with arms outstretched and held together above the head
4×6 rep	@1’	Push from the bottom of the pool and perform vertical kicks	4×6 rep	@1’	Push from the bottom of the pool and perform vertical kicks
4×15 m	@1’	From the 5-m mark to the 20-m mark (without push-off) perform undulatory underwater swimming	4×15 m	@1’	From the 5-m mark to the 20-m mark (without push-off) perform undulatory underwater swimming
4×6 rep	@1’	Vertical kicks with arms outstretched and held together above the head	4×10 rep each leg	@2’30”	Downbeat action simulation (i.e., hip flexion + knee extension) – 4 weights (moment of inertia: 738.86 kg/cm^2^)
4×6 rep	@1’	Push from the bottom of the pool and perform vertical kicks			
4×15 m	@1’	From the 5-m mark to the 20-m mark (without push-off) perform undulatory underwater swimming			

@: start every “X” time

**Table:** 

SESSION 14, Wednesday, Week 5					
**Only Water (WO)**			**Water + Dry-land (WD)**		
Set	Rest	Exercise	Set	Rest	Exercise
1×12 rep	@1’15”	Competition between swimmers. In pairs, reach 12.5 m using undulatory underwater swimming before your teammate	1×12 rep	@1’15”	Competition between swimmers. In pairs, reach 12.5 m using undulatory underwater swimming before your teammate
1×12 rep	@1’15”	Competition between swimmers. In pairs, reach 12.5 m using undulatory underwater swimming before your teammate	4×10 rep each leg	@2’30”	Upbeat action simulation (i.e., hip extension + knee flexion) – 4 weights (moment of inertia: 738.86 kg/cm^2^)

@: start every “X” time

## References

[ref1] Arellano, R., Pardillo, S. & Gavilán, A. (2002). Underwater undulatory swimming: kinematic characteristics, vortex generation and application during the start, turn and swimming strokes. Proceedings of the XXth International Symposium on Biomechanics in Sports, 29–41.

[ref2] Arellano, R., Ruiz-Navarro, J. J., Barbosa, T. M., López-Contreras, G., Morales-Ortíz, E., Gay, A., López-Belmonte, Ó., González-Ponce, Á. & Cuenca-Fernández, F. (2022). Are the 50 m Race Segments Changed From Heats to Finals at the 2021 European Swimming Championships? Frontiers in Physiology, 13, 1–24. 10.3389/fphys.2022.797367 PMC9326221 35910554

[ref3] Atkinson, G. & Reilly, T. (1996). Circadian variation in sports performance. Sports Medicine, 21(4), 292–312. 10.2165/00007256-199621040-00005 8726347

[ref4] Atkison, R. R., Dickey, J. P., Dragunas, A., & Nolte, V. (2014). Importance of sagittal kick symmetry for underwater dolphin kick performance. Human Movement Science, 33, 298–311. 24290609 10.1016/j.humov.2013.08.013

[ref5] Balsalobre-Fernández, C., Glaister, M. & Lockey R. A. (2015). The validity and reliability of an iPhone app for measuring vertical jump performance. Journal of Sports Sciences, 33(15), 1574–1579. 10.1080/02640414.2014.996184 25555023

[ref6] Bielec, G., Makar, P., Laskowski, R., Olek, R. A. (2013). Kinematic variables and blood acid-base status in the analysis of collegiate swimmers' anaerobic capacity. Biology of Sport, 30 (3), 213-217. 10.5604/20831862.1059303. 24744491 PMC3944568

[ref7] Bielec, G., Makar, P. (2010). Variability in swimmers' individual kinematics parameters versus training loads. Biology of Sport, 27 (2), 143-147. 10.5604/20831862.913082.

[ref8] Cohen, J. (1988). Statistical power analysis for the behavioural sciences (pp. 20–27). Lawrence Erlbaum Associates.

[ref9] Collard, L., Gourmelin, E. & Schwob, V. (2013). The fifth stroke: the effect of learning the dolphin-kick technique on swimming speed in 22 novice swimmers. Journal of Swimming Research, 21(1), 1–15.

[ref10] Connaboy, C., Coleman, S., Moir, G. & Sanders, R. (2010). Measures of reliability in the kinematics of maximal undulatory underwater swimming. Medicine and Science in Sports and Exercise, 42(4), 762–770. 10.1249/MSS.0b013e3181badc68 19952849

[ref11] Connaboy, C., Naemi, R., Brown, S., Psycharakis, S., McCabe, C., Coleman, S. & Sanders, R. (2016). The key kinematic determinants of undulatory underwater swimming at maximal velocity. Journal of Sports Sciences, 34(11), 1036–1043. 10.1080/02640414.2015.1088162 26367778

[ref12] Crespo, E., Ruiz-Navarro, J. J., Cuenca-Fernández, F. & Arellano, R. (2021). Post-eccentric flywheel underwater undulatory swimming potentiation in competitive swimmers. Journal of Human Kinetics, 79(1), 145–154. 10.2478/hukin-2021-0068 34400994 PMC8336562

[ref13] Cuenca-Fernández, F., Ruiz-Navarro, J. J., Polach, M., Arellano, R. & Born D. P. (2022). Turn Performance Variation in European Elite Short-Course Swimmers. International Journal of Environmental Research and Public Health, 19(9), 1–11. 10.3390/ijerph19095033 PMC9102928 35564428

[ref14] Elipot, M., Dietrich, G., Hellard, P. & Houel, N. (2010). Cinalysis: A new software for swimming races analysis. *8th Conference of the International Sports Engineering Association (ISEA)*. 10.1016/j.proeng.2010.04.191

[ref15] García-Ramos, A., Tomazin, K., Feriche, B., Strojnik, V., De La Fuente, B., Argüelles-Cienfuegos, J., Strumbelj, B. & Štirn, I. (2016). The relationship between the lower-body muscular profile and swimming start performance. Journal of Human Kinetics, 50(1), 157–165. 10.1515/hukin-2015-0152 28149353 PMC5260650

[ref16] Gonjo, T. & Olstad, B. H. (2021). Race analysis in competitive swimming: A narrative review. International Journal of Environmental Research and Public Health, 18(1), 1–16. 10.3390/ijerph18010069 PMC7795652 33374118

[ref17] Helmy, A. (2013). The effects of combined program (land-and aquatic exercises) on gliding underwater for young swimmers. Science, Movement & Health, 13(2), 118–123.

[ref18] Higgs, A. J., Pease, D. L. & Sanders, R. H. (2017). Relationships between kinematics and undulatory underwater swimming performance. Journal of Sports Sciences, 35(10), 995–1003. 27431482 10.1080/02640414.2016.1208836

[ref19] Lyakh V., Mikołajec K., Bujas P., Litkowycz R. (2014). Review of Platonov's sports Training Periodization. General Theory and its Practical Application-Kiev: Olympic Literature, 2013 (part two). Journal of Human Kinetics, 46(1), 273–278. 10.2478/hukin-2014-0131 PMC4327377 25713686

[ref20] Lyakh V., Mikolajec K., Bujas P., Witkowski Z., Tomasz Zając T., Litkowycz R.,2 & Banys, D. (2016). Periodization in Team Sport Games-A Review of Current Knowledge and Modern Trends in Competitive Sports. Journal of Human Kinetics, 54 (1), 173-180. 10.1515/hukin-2016-0053

[ref21] Mason, B. R. & Cossor, J. M. . (2001). Swim turn performances at the Sydney 2000 Olympic Games. In J. Blackwell & R. H. Sanders (Eds.), *Proceedings of swim sessions: XIX international symposium on biomechanics in sports* (pp. 65–69).

[ref22] Mckay, A. K. A., Stellingwerff, T., Smith, E. S., Martin, D. T., Mujika, I., Goosey-tolfrey, V. L., Sheppard, J. & Burke, L. M. (2022). Defining training and performance caliber : a participant classification framework. International Journal of Sports Physiology and Performance, 17, 317–331. 34965513 10.1123/ijspp.2021-0451

[ref23] McLeod, I. (2009). Swimming anatomy. Human Kinetics.

[ref24] Mookerjee, S., Bibi, K. W., Kenney, G. A. & Cohen, L. (1995). Relationship between isokinetic strength, flexibility, and flutter kickking speed in female collegiate swimmers. Journal of Strength and Conditioning Research, 9(2), 71–74.

[ref25] Mujika, I., Busso, T., Lacoste, L., Barale, F., Geyssant, A. & Chatard, J.-C. (1996). Modeled responses to training and taper in competitive swimmers. Medicine and Science in Sports and Exercise, 28(2), 251–258. 8775162 10.1097/00005768-199602000-00015

[ref26] Naemi, R. & Sanders, R. H. (2008). A “hydrokinematic” method of measuring the glide efficiency of a human swimmer. Journal of Biomechanical Engineering, 130(6), 1–9. 10.1115/1.3002764 19045545

[ref27] Navarro, F., Oca, A. & Castañón, F. J. (2003). The young swimmer’s training [El entrenamiento del nadador joven]. Gymnos.

[ref28] Nikolaidis, P. T. (2012). Age-and sex-related differences in force-velocity characteristics of upper and lower limbs of competitive adolescent swimmers. Journal of Human Kinetics, 32(1), 87–95. 10.2478/v10078-012-0026-4 23487511 PMC3590871

[ref29] Papic, C., Sanders, R. H., Naemi, R., Elipot, M. & Andersen, J. (2020). Improving data acquisition speed and accuracy in sport using neural networks. Journal of Sports Sciences, 00(00), 1–10. 10.1080/02640414.2020.1832735 33140693

[ref30] Perez-Olea, J. I., Valenzuela, P. L., Aponte, C. & Izquierdo, M. (2018). Relationship between dryland strength and swimming performance: pull-up mechanics as a predictor of swimming speed. Journal of Strength and Conditioning Research, 32(6), 1637–1642. 29786624 10.1519/JSC.0000000000002037

[ref31] Ruiz-Navarro, J. J., Andersen, J. T., Cuenca-Fernández, F., López-Contreras, G., Morouço, P. G. & Arellano, R. (2022a). Quantification of swimmers ’ ability to apply force in the water : the potential role of two new variables during tethered swimming. Sports Biomechanics, 00(00), 1–13. 10.1080/14763141.2022.2089220 35714061

[ref32] Ruiz-Navarro, J. J., Cano-Adamuz, M., Andersen, J. T., Cuenca-Fernández, F., López-Contreras, G., Vanrenterghem, J. & Arellano, R. (2021). Understanding the effects of training on underwater undulatory swimming performance and kinematics. Sports Biomechanics, 00(00), 1–16. 10.1080/14763141.2021.1891276 33663350

[ref33] Ruiz-Navarro, J. J., Cuenca-Fernández, F., Sanders, R. & Arellano, R. (2022b). The determinant factors of undulatory underwater swimming performance: A systematic review. Journal of Sports Sciences, 40(11), 1243–1254. 10.1080/02640414.2022.2061259 35384796

[ref34] Ruiz-Navarro, J. J., Gay, A., Cuenca-Fernández, F., López-Belmonte, Ó., Morales-Ortíz, E., López-Contreras, G. & Arellano, R. (2022c). The relationship between tethered swimming , anaerobic critical velocity , dry-land strength , and swimming performance performance. International Journal of Performance Analysis in Sport, 22(3), 407–421. 10.1080/24748668.2022.2072561

[ref35] Ruiz-Navarro, J. J., López-Belmonte, Ó., Gay, A., Cuenca-Fernández, F. & Arellano, R. (2022d). A new model of performance classification to standardize the research results in swimming. European Journal of Sport Science, 23(4), 478–488. 35193458 10.1080/17461391.2022.2046174

[ref36] Ruiz-Navarro, J. J., Morouço, P. G. & Arellano, R. (2020). Relationship between tethered swimming in a flume and swimming performance. International Journal of Sports Physiology and Performance, 15(8), 1087–1094. 10.1123/ijspp.2019-0466 32032941

[ref37] Shimojo, H., Sengoku, Y., Miyoshi, T., Tsubakimoto, S. & Takagi, H. (2014). Effect of imposing changes in kick frequency on kinematics during undulatory underwater swimming at maximal effort in male swimmers. Human Movement Science, 38, 94–105. 10.1016/j.humov.2014.09.001 25278097

[ref38] Tanaka, T., Hashizume, S., Kurihara, T., & Isaka, T. (2022). The Large and Strong Vortex Around the Trunk and Behind the Swimmer is Associated with Great Performance in Underwater Undulatory Swimming. Journal of Human Kinetics, 84, 64–73. 10.2478/hukin-2022-0087. 36457469 PMC9679196

[ref39] Ungerechts, B. E., Persym, U. & Colman, V. (2000). Analysis of swimming techniques using vortex traces. XVIII International Symposium on Biomechanics in Sports. ISBS - Conference Proceedings Archive. https://ojs.ub.uni-konstanz.de/cpa/article/view/2532 (accessed on 01/12/2022).

[ref40] Vaughan, C. L. (1982). Smoothing and differentiation of displacement-time data: An application of splines and digital filtering. International Journal of Bio-Medical Computing, 13(5), 375–386. 10.1016/0020-7101(82)90003-4 6897057

[ref41] Veiga, S., Lorenzo, J., Trinidad, A., Pla, R., Fallas-campos, A. & Rubia, A. De. (2022). Kinematic analysis of the underwater undulatory swimming cycle : a systematic and synthetic review. International Journal of Environmental Research and Public Health, 19, 1–26. 10.3390/ijerph191912196 PMC9566274 36231498

[ref42] Veiga, S. & Roig, A. (2016). Underwater and surface strategies of 200 m world level swimmers. Journal of Sports Sciences, 34(8), 766–771. 10.1080/02640414.2015.1069382 26186108

[ref43] Veiga, S., Qiu, X., Trinidad, A., Dolek, B. E., De la Rubia, A., & Navarro, E. (2024). Effect of the Skill, Gender, and Kick Order on the Kinematic Characteristics of Underwater Undulatory Swimming in the Dorsal Position. Journal of Human Kinetics, 90, Ahead of print. 10.5114/jhk/168600 PMC10875688 38380311

[ref44] Vennell, R., Pease, D. & Wilson, B. (2006). Wave drag on human swimmers. Journal of Biomechanics, 39(4), 664–671. 10.1016/j.jbiomech.2005.01.023 16439236

[ref45] Willems, T. M., Cornelis, J. A. M., De Deurwaerder, L. E. P., Roelandt, F. & De Mits, S. (2014). The effect of ankle muscle strength and flexibility on dolphin kick performance in competitive swimmers. Human Movement Science, 36, 167–176. 24984154 10.1016/j.humov.2014.05.004

[ref46] Yamakawa, K. K., Shimojo, H., Takagi, H. & Sengoku, Y. (2022). Changes in kinematics and muscle activity with increasing velocity during underwater undulatory swimming. Frontiers in Sports and Active Living, 4, 1–12. 10.3389/fspor.2022.829618 PMC9051435 35498520

[ref47] Zamparo, P., Pendergast, D. R., Termin, B. & Minetti, A. E. (2002). How fins affect the economy and efficiency of human swimming. Journal of Experimental Biology, 205(17), 2665–2676. 12151372 10.1242/jeb.205.17.2665

